# Genetic Insights into the Middle East Respiratory Syndrome Coronavirus Infection among Saudi People

**DOI:** 10.3390/vaccines9101193

**Published:** 2021-10-17

**Authors:** Hatem A. Abuelizz, Maha M. AlRasheed, Ali Alhoshani, Tariq Alhawassi

**Affiliations:** 1 Department of Pharmaceutical Chemistry, College of Pharmacy, King Saud University, P.O. Box 2457, Riyadh 11451, Saudi Arabia; 2 Department of Clinical Pharmacy, College of Pharmacy, King Saud University, P.O. Box 2457, Riyadh 11451, Saudi Arabia; mahalrasheed@ksu.edu.sa (M.M.A.); tarriq@ksu.edu.sa (T.A.); 3 Department of Pharmacology and Toxicology, College of Pharmacy, King Saud University, P.O. Box 2457, Riyadh 11451, Saudi Arabia; ahoshani@ksu.edu.sa

**Keywords:** dipeptidyl peptidase 4, DPP4, MERS-CoV, middle east respiratory syndrome

## Abstract

Background: The Middle East respiratory syndrome coronavirus (MERS-CoV) was isolated for the first time in Saudi Arabia from a patient suffering from atypical pneumonia. The Saudi Genome database was built by King Abdulaziz Medical City via the next-generation sequencing of 7000 candidates. Method: A large list of point mutations were reported in the region of the dipeptidyl peptidase 4 (DPP4) gene. The DPP4 amino acid residues correlated to MERS-CoV entry and the site of activity of DPP4 inhibitors was investigated. We retrieved the SNPs (Single-Nucleotide Polymorphism) with a variation frequency of >0.05. Results: SNP 2:162,890,175 and SNP 2:162,891,848 in the intronic region were located within 50 bp of amino acid residues responsible for MERS-CoV entry, amino acids 259–296 and 205–258, respectively. The variation frequency of SNP 2:162,890,175 was 2321 out of 2379 screened individuals. Moreover, mutation of SNP 2:162,891,848, which is located near amino acid residues E205 and E206 (crucial for the activity of DPP4 inhibitors), occurred in 76 out of 2379 screened individuals. Conclusions: Our study shows high variation frequency in the DPP4 region reported in the Saudi Genome database. The identified SNPs are of high significance for MERS-CoV infection in better understanding disease pathogenesis.

## 1. Introduction

In 2012, the first Middle East Respiratory Syndrome Coronavirus (MERS-CoV) was isolated in Saudi Arabia from a patient suffering from atypical pneumonia [1,2]. The virus is characterized as a single-stranded RNA enveloped virus and belongs to a family called Coronaviridae (Betacoronavirus genus) which is of the Nidovirales order [3]. Since the discovery of MERS-CoV, more than 2519 human infections have been described (as of January 2020), 34.3% (866 patients) of which were fatal [4]. Although camels have been identified as reservoir hosts and have only shown common cold symptoms, they remain the main source of infection. The transmission of the disease to humans has occurred either from close contact with the animal or via its contaminated products [5,6]. The fatality rate and easy transmissibility from the animal reservoir to humans make it a serious public health threat. Individuals with diabetes, renal, or respiratory diseases are most susceptible to MERS-CoV pneumonia, and have a fatality rate as high as 40% [7].

The novel infectious disease that emerged in 2019 and rapidly became a worldwide pandemic is caused by a novel betacoronavirus [8]. The sudden spread and emergence of the coronavirus, currently known as COVID-19, is very similar to SARS, the severe acute respiratory syndrome, another epidemic caused by a coronavirus that originated in China in 2002 [9]. Nearly a decade later MERS-CoV erupted in the Middle East. The dipeptidyl peptidase 4 (DPP4) is the cellular receptor for MERS-CoV, and on the other hand, SARS-CoV-2 enters host cells by binding to angiotensin-converting enzyme 2 (ACE2) [10].

DPP4 is a serine protease enzyme involved in the cleavage and regulation of the glucagon-like peptide-1 (GLP-1) and glucose-dependent insulinotropic polypeptide (GIP). They constitute some of the most critical therapeutic targets for the treatment of type 2 diabetes mellitus [11,12]. DPP4 has been characterized as a 110 kDa plasma membrane glycoprotein. The DPP4 gene is localized near the GLP-1 and GLP-2 encoding genes, consisting of 26 exons ranging between 45 bases to 1.4 kilobases. The encoded protein exists as an asymmetric dimer, each monomer consisting of 766 amino acids [13].

Genetic factors were considered for a long time to play a key role in explaining susceptibility variation. Genes are the variables involved in immune responses and susceptibility to infectious disease pressure. Furthermore, the variation in the outcome following infections provides a significant insight into the genetic variability of the immune response and predisposition. Genome-wide sequencing as whole-exome or whole-genome is an important diagnostic tool that can be used to predict the sensitivity and specificity of the disease [14]. Moreover, naturally occurring polymorphisms have been identified in DPP4. The identified polymorphism has a negative impact on the MERS-CoV cellular entry and the development of the disease [15]. This has raised the question of how much of the variation in MERS-CoV-correlated genes have been demonstrated in the population and might influence the infection susceptibility. Infection susceptibility toward MERS-CoV has been correlated to the affinities of the DPP4 receptor to the viral spike proteins. Moreover, multiple glycosylation sites have been found on the DPP4 receptor and are considered crucial for the virus entry and susceptibility [16]. The genetic cataloging of human genetic risk factors can influence the treatment and prevention of MERS-CoV [17]. Therefore, we aim to identify the genetic variations of the required genes for the MERS-CoV infection among the Saudi population.

The Saudi Genome Database was built by King Abdulaziz Medical City employing next-generation sequencing for 2357 candidates, initially to understand the mendelian disease suspected mutations [18]. Since then, the database has been expanded to include unrelated candidates to reach a total of 7000 candidates. A large list of point mutations have been reported by the database in the region of DPP4. A variety of the DPP4 point mutations are showing high prevalence in the Saudi population [19]. In the current study, we investigated the amino acid residues related to the virus entry, putative N-glycosylation sites, and the DPP4 inhibitor site of activity. We screened for crucial SNPs in the DPP4 region that can also be targeted for studying susceptibility and prevention of MERS-CoV infection.

## 2. Materials and Methods

### 2.1. Data Collection

SNP screening in the DPP4 gene was carried out using the setup of the Saudi Genome Project [18,19]. The screening was based on the commercial Inherited Disease Panel of LifeTech using the Ion Torrent sequencing technology. The panel consisted of 3085 known disease genes. Using primer-based sequencing, the whole-exome sequencing was performed targeting 25,000 genes and open-reading frames. The annotation of significant point mutations was performed in the DPP4 gene region located on chromosome 2, between SNPs number 162,848,808 and 162,930,975.

### 2.2. Data Processing and Analysis

The frequent variants reported in the study were nominated by the Human Gene Mutation Database as disease-mutation for mendelian disease [19]. The variant occupation involves the variation detection and filtration has not included the variants unrelated to the disease. The intronic variants with more than 20 bases distance from the exonic terminals were excluded. In the public databases, the variants with a minor allele frequency of >1% were excluded. Based on a score of >100 computed by Torrent Suite, the filtration of the remaining variants was carried out according to the confidence of base depth, calls, and context bases. The variants considered to be homozygous were filtered. The remaining variants were then sent to the Sanger sequencing to validate the variants.

### 2.3. Annotation

We called all the reported SNPs in the Saudi Genome database (https://www.saudigenomeproject.com accessed on 1 November 2020) and retrieved SNPs with a variation frequency of >0.05. We determined the SNPs in the exonic and intronic regions. The translation of the amino acid residues of interest to their corresponding SNP location was performed using the University of California Santa Cruz Genome Browser (http://genome.ucsc.edu accessed on 1 November 2020). Conversely, the SNPs with a high variation frequency were correlated to the amino acid residues on the DPP4 protein. SNPs located within 50 bp upstream and downstream of the amino acid residues of interest were also identified.

## 3. Results

We collated all the DPP4-related mutations listed in the Saudi Genome Database. The database included genetic analysis of more than 9000 patients identified with mendelian disease. This was including variants of uncertain significance, as well as, variants with a significance of more than 0.05. The Saudi Human Genome Program has classified many variants as homozygous and demonstrated them for their significance in the DPP4 region. The SNPs collection has included SNPs responsible for the virus entry, DPP4 inhibitors sites, and DPP4 glycosylation sites.

### 3.1. High-Frequency SNP Variation

The DPP4 region is located on chromosome 2, between the 162,846,755 and 162,932,725 loci. Within this region, 249 variations were reported in the Saudi Genome database. Among the reported variations, 10 SNPs exhibited a variation frequency of >0.05 in the intronic and exonic regions (Table 1). Eight of these 10 SNPs were in the DPP4 introns and two were in the exons. SNP 2:162,890,175 in the intronic region was within 50 bp of the amino acid residues at positions 259–296 in the DPP4 protein. Moreover, SNP 2:162,891,848 was within 50 bp of the amino acid residues at positions 205–258 in DPP4. In the exonic region, SNPs 2:162,929,979 and 2:162.930,725 were correlated to the L8 amino acid in the DPP4 protein and exon 1 of the DPP4 gene, respectively. The mutation corresponding to SNP 2:162,929,979 caused an amino acid change from leucine to glutamic acid.

### 3.2. Amino Acid Residues Crucial for MERS-CoV Entry

The interactions between the virus spike protein (S) and DPP4 for MERS-CoV entry have been identified using crystallography. The S protein crystallography has revealed 15 amino acid residues in DPP4 that constituted the site of interaction. The interactive amino acid residues with the S protein were in the cysteine-rich and glycosylation-rich domains (K267, F269, Q286, T288, A289, A291, L294, I295, H298, R317, Y322, R336, Q344, I346, and K392) [20]. The SNP locations of the amino acid residues have been identified (Table 2). The genes encoding the two amino acid residues A289 and K392 were reported in the Saudi Genome database to have mutations among the Saudi population. Those mutations did not affect the translation of the gene to the corresponding amino acid. The genes encoding the other amino acid residues responsible for the virus entry did not show any mutations in the database. However, two amino acids were located within 50 bp of highly frequent variants in the Saudi population.

### 3.3. Amino Acid Residues as Sites for N-Glycosylation

The major factor impacting DPP4 as the site of MERS-CoV binding was found to be its glycosylation state. The glycosylation of DPP4 is species-specific and impacts the MERS-CoV host range [21]. We examined nine asparagine residues that have been identified as sites of N-linked glycosylation (N85, N95, N150, N219, N229, N281, N321, N520, and N680) (Table 3) [22]. Among them, N219 was reported in the Saudi Genome database to have a variation frequency of 0.00042. The observed mutation has a putative effect on the translation of the amino acid to residue into lysine. Moreover, it was located within 50 bp of SNP 2:162,891,779, which had the same variation frequency of 0.00042. Moreover, six amino-acid-correlated SNPs were located within 50 bp of a reported mutation in the database. None of these SNPs had variation frequencies >0.05.

## 4. Discussion

In this study, we evaluated the high variation frequency in the DPP4 region reported in the Saudi Genome database. Moreover, we have studied the impact of the reported variations in the intronic region of the gene and the encoded protein. Among the reported SNPs, one mutation at SNP 2:162,929,979 has shown to have a potential effect on the amino acid translation from leucine to glutamic acid. It has been seen to occur in 1572 variants of 2379 screened patients (Table 1). This variation is located within the exonic region of the gene encoding the amino acids 3 to 25. To our knowledge, no relevance has yet been allotted to this site for the DPP4 activity or virus entry. On the other hand, one mutation with high variation frequency is located within 50 bp of the amino acid residues believed to be important for the virus entry. The SNP 2:162,890,175 is located near the region translating the amino acid residues K267, F269, Q286, T288, A289, A291, L294, and I295 (Table 2). Other mutations were detected for SNPs 2:162,890,071 and 2:162,877,091, related to the amino acid residues A289 and K392, respectively (Table 2). However, these mutations were of low variation frequencies.

As reported earlier, MERS-CoV does not bind to the mouse DPP4 (mDPP4). The two key amino acid residues in the mouse are R288 and T330, equivalent to L294 and R334 in human DPP4 (hDPP4). These amino acid residues in the mouse do not allow virus entry. However, the binding of MERS-CoV to DPP4 in the mouse has occurred in the presence of the mutation related to the two amino acids (A288L and T330R) in hDPP4 [17]. In our study, we have shown that the variation frequency of SNP 2:162,890,175 occurred in the 2321 variants of 2379 screened patients (Table 2). This mutation has a potential effect on the translation of the residue L294 that is thought to play an important role in MERS-CoV susceptibility.

Although the glycosylation sites included in our study were not essential for the DPP4 activity, its glycosylation states have been linked to virus susceptibility [22,23]. The permissibility of MERS-CoV to different host species is governed by the variable DPP4 glycosylation [23]. Among the nine residues in the DPP4 region, the N219, N229, and N281 have been identified as mutation sites in the Saudi population. The SNP 2:162,891,792 mutation from N to A affects the N219 translation to lysine (Table 3). Furthermore, the SNP 2:162,891,848 is located within 50 bp of the above three residues, whereby the mutation at the SNP locus occurred in the 76 variants of 2379 screened patients.

Although the site of entry of MERS-CoV (DPP4) is different to that of SARS-CoV2 (ACE2), the modeling studies have suggested that DPP4 could be a coreceptor for SARS-CoV-2 viral entry and disease severity [24,25]. The studies indicated DPP4 dysregulation and late inflammatory immune response caused by lung injury among COVID-19 patients [24,26]. It has been observed that DPP4 inhibitors, including sitapliptin, might be used for the prevention or minimizing of the risk and severity of COVID-19 infection [24,27,28,29]. On the other hand, the clinical risk factors associated with the MERS-CoV infection have been identified in our previous study. It was found that among 348 cases confirmed by RT-PCR, diabetes was a risk factor for MERS-CoV by 22% (*p*-value = 0.02). None of the identified diabetic patients on DPP4 inhibitors were infected by the disease [30]. The receptor binding studies of the DPP4 inhibitors have identified important residues (E205 and E206) for their inhibition activity [31]. The residues E205 and E206 are located within 50 bp of SNP 2:162,891,848. This SNP has shown high variation frequency among the Saudi population (Table 1). Hence, this mutation may provide insights into the function of DPP4 inhibitors in minimizing severity and susceptibility to coronavirus infection.

## 5. Conclusions

Various genetic patterns help to develop an understanding and prevention of the risk of infection, immune response to the virus, disease severity, and death. Our study has provided some genetic insight into MERS-CoV infection susceptibility and prevention in the Saudi population. We demonstrated that SNP 2:162,891,848 is of high significance for MERS-CoV infection susceptibility. This SNP is located within 50 bp of the amino acid residues that play an important role in the virus entry as well as glycosylation of the DPP4. Moreover, DPP4 inhibitors may play an important role in reducing disease severity among MERS-CoV patients. This study provides further insights into the pathogenesis of the virus and may inspire further research on therapeutic targets and prevention of MERS-CoV infection.

## Figures and Tables

**Table 1:** SNPs loci and frequencies with observed mutation and their correlated amino acids.

SNP Loci	Observed Mutation	Variation Frequency	Variation Frequency (Homozygous)	Site of SNP	Correlated Amino Acid Residue	Correlated Amino Acid Residue within 50 bp
2:162,873,188	T → C	0.13	0.09	Intron	Not applicable	Not applicable
2:162,877,028	T → A	0.24	0.05	Intron	Not applicable	Not applicable
2:162,879,452	T → C	0.22	0.05	Intron	Not applicable	Not applicable
2:162,890,175	T → C	0.62	0.36	Intron	Not applicable	259–296
2:162,890,217	G → A	0.33	0.07	Intron	Not applicable	Not applicable
2:162,891,848	C → T	0.78	0.42	Intron	Not applicable	205–258
2:162,894,766	A → G	0.26	0.06	Intron	Not applicable	Not applicable
2:162,929,732	G → A	0.05	0.02	Intron	Not applicable	Not applicable
2:162,929,979	A → G	0.53	0.13	Exon	L8Leu to Glu	3–25
2:162.930,725	T → G	0.07	0.06	Exon	Not applicable	Not applicable

**Table 2:** Amino acids crucial for the virus entry with observed mutation and their correlated SNPs.

Amino Acid Residue	SNP Loci	Observed Mutation	Variation Frequency	Variation Frequency (Homozygous)	Number of SNPs with a Variation Frequency <0.05 Detected within 50 bp	Number of SNPs with a Variation Frequency >0.05 Detected within 50 bp
K267	2:162,890,137	---	---	---	5	1
F269	2:162,890,131	---	---	---	5	1
Q286	2:162,890,080	---	---	---	3	Not available
T288	2:162,890,074	---	---	---	3	Not available
A289	2:162,890,071	A → G	0.00042	0	2	Not available
A291	2:162,890,056	---	---	---	2	Not available
L294	2:162,890,053	---	---	---	2	Not available
H298	2:162,881,443	---	---	---	1	Not available
R317	2:162,881,386	---	---	---	Not available	Not available
Y322	2:162,881,371	---	---	---	Not available	Not available
R336	2:162,881,329	---	---	---	1	Not available
Q344	2:162,879,301	---	---	---	1	Not available
I346	2:162,879,295	---	---	---	1	Not available
K392	2:162,877,091	T → deletion	0.00126	0	5	Not available

**Table 3:** N-glycosylation amino acids, SNP loci and their variation frequencies.

Amino Acid Residue	SNP Loci	Observed Mutation	Variation Frequency	Variation Frequency (Homozygous)	Near Variations Detected within 50 bp
N85	2:162,903,459	Not available	---	---	Variation C→T at 2:162,903,410
N92	2:162,903,429	Not available	---	---	Variation C→T at 2:162,903,410
N150	2:162,903,264	Not available	---	---	Variation T→A at 2:162,903,214
N219	2:162,891,792	G → A	0.00042	0	Variation A→deletion at 2:162,891,779
N229	2:162,891,764	Not available	---	---	None
N281	2:162,890,098	Not available	---	---	Variation C→G at 2:162,890,108
N321	2:162,881,378	Not available	---	---	None
N520	2:162,873,289	Not available	---	---	Variation G→A at 2:162,873,306
N685	2:162,851,884	Not available	---	---	None

## Data Availability

Not applicable.
